# 150. Updated Clinical Guidelines for Treatment and Prophylaxis of Plague

**DOI:** 10.1093/ofid/ofab466.150

**Published:** 2021-12-04

**Authors:** Christina Nelson, Katharine Cooley, Shannon Fleck-Derderian

**Affiliations:** 1 Centers for Disease Control and Prevention, Fort Collins, CO; 2 Centers for Disease Control and Prevention/Division of Vector-Borne Diseases/Alaka’ina Foundation of Companies , Fort Collins, CO

## Abstract

**Background:**

Plague still occurs naturally in the western United States, Latin America, Asia, and Africa. *Yersinia pestis*, the causative agent of plague, is a Tier 1 bioterrorism agent due to its potential for aerosol release and high fatality rates. Recommendations for treatment and post-exposure prophylaxis (PEP) of plague were published in 2000 and included limited first-line options for treating plague, namely streptomycin or gentamicin. Doxycycline or ciprofloxacin were recommended for PEP. However, since 2000 new human clinical data and animal data have become available, and the FDA has approved additional antimicrobials for plague.

**Methods:**

CDC developed updated, evidence-based guidelines for treatment and prophylaxis of plague using a comprehensive process. To collect evidence on relative efficacy of various antimicrobials for treatment of plague, the guidelines team conducted systematic literature reviews and analyzed U.S. surveillance data. Results of these investigations were published in *Clinical Infectious Diseases* in 2020. We also hosted several meetings with subject matter experts and clinical organizations (IDSA, AAP, etc.), federal agencies, and others to review relevant data and gather individual input on treatment and prophylaxis of plague.

**Results:**

The forthcoming plague guidelines will include several important updates. First-line treatment options have been expanded to include ciprofloxacin, levofloxacin, and moxifloxacin in addition to streptomycin and gentamicin. For PEP, levofloxacin and moxifloxacin are now first-line options in addition to doxycycline and ciprofloxacin. Trimethoprim-sulfamethoxazole is now one of several new alternative options for PEP. The updated guidelines also include recommendations for treatment of clinical forms of plague other than pneumonic. Additional special populations such as immunocompromised persons and neonates are also covered.

**Conclusion:**

Plague remains a threat, both as a naturally occurring disease and as a potential bioterrorism weapon, and preparedness and early recognition are key to effective response. The updated clinical guidelines will be a useful tool for clinicians to manage antimicrobial treatment and PEP for plague.

**Disclosures:**

**All Authors**: No reported disclosures

